# P-166. Susceptibility to Measles among immigrants in South Korea

**DOI:** 10.1093/ofid/ofae631.371

**Published:** 2025-01-29

**Authors:** Ran Lee, Jang Gwan Yoon, A Ram Park, So Hyun Bae, Jun Hwi Cho, Jin Kim, Kyung-Hwa Park, Seong Woo Choi, Bong Kyu Sun, Kyung Hak Kim, Sun Seog Kweon, Seong Eun Kim

**Affiliations:** Gwangju Center for Infectious Diseases Control and Prevention, seo-gu, Kwangju-jikhalsi, Republic of Korea; Gwangju Center for Infectious Diseases Control and Prevention, seo-gu, Kwangju-jikhalsi, Republic of Korea; Gwangju Center for Infectious Diseases Control and Prevention, seo-gu, Kwangju-jikhalsi, Republic of Korea; Gwangju Center for Infectious Diseases Control and Prevention, seo-gu, Kwangju-jikhalsi, Republic of Korea; Gwangju Center for Infectious Diseases Control and Prevention, seo-gu, Kwangju-jikhalsi, Republic of Korea; Gwangju Center for Infectious Diseases Control and Prevention, seo-gu, Kwangju-jikhalsi, Republic of Korea; Chonnam National University Medical School, GwangJu, Kwangju-jikhalsi, Republic of Korea; Department of Preventive Medicine, Chosun University Medical School, Seo-gu, Kwangju-jikhalsi, Republic of Korea; Center for Global Diaspora Studies, Chonnam National University, Seo-gu, Kwangju-jikhalsi, Republic of Korea; Center for Global Diaspora Studies, Chonnam National University, Seo-gu, Kwangju-jikhalsi, Republic of Korea; Gwangju Institute for Public Health and Equity, Nam-gu, Kwangju-jikhalsi, Republic of Korea; Chonnam National University Medical School, GwangJu, Kwangju-jikhalsi, Republic of Korea

## Abstract

**Background:**

Despite the World Health Organization's declaration of measles elimination in Korea in 2014, the country continues to encounter outbreaks, predominantly from cases imported via international travel. This study investigates the susceptibility to measles among immigrants residing in Korea, with an emphasis on their immunity status.

**Methods:**

Conducted in Gwangju, Republic of Korea, this research measured measles immunoglobulin G (IgG) serum levels among immigrants from various nationalities.

**Results:**

The study enrolled 415 participants, predominantly from Vietnam (18.8%), Uzbekistan (17.1%), Kazakhstan (10.4%), and the Russian Federation (10.1%). Overall, 82.2% exhibited seropositivity for measles IgG. Individuals under 30 years showed significantly lower seroprevalence at 61.8%, compared to 90.8% in those aged 30 and above. The lowest seroprevalences were noted in Cambodia (64.9%), the Russian Federation (69.8%), Ukraine and Mongolia (71.4% each), Nepal (75.0%), Kazakhstan (76.7%), and Uzbekistan (86.5%). The countries with the largest gaps from WHO/UNICEF published MCV2 coverage rates were Russian Federation and Mongolia (average MCV2 coverage rate 95.9% and 95.5% from 2000 to 2022, respectively). There were also significant disparities in measles immunity based on visa types, with work visa holders showing considerably lower immunity (75%) compared to those with residence visas (87.2%; Chi-square test, *P*=0.02).

**Conclusion:**

The results highlight significant disparities in measles immunity among immigrants, particularly according to age, countries, and visas. The study underscores the critical need for targeted vaccination strategies and enhanced surveillance in Korea. Implementing tailored catch-up vaccination programs is essential to reduce the risk of measles outbreaks within this vulnerable population.

**Disclosures:**

**All Authors**: No reported disclosures

